# Population Genomic Screening for Genetic Etiologies of Neurodevelopmental/Psychiatric Disorders Demonstrates Personal Utility and Positive Participant Responses

**DOI:** 10.3390/jpm11050365

**Published:** 2021-05-01

**Authors:** Karen E. Wain, Kasia Tolwinski, Emily Palen, Alexis R. Heidlebaugh, Karahlyn Holdren, Lauren Kasparson Walsh, Matthew T. Oetjens, David H. Ledbetter, Christa Lese Martin

**Affiliations:** 1 Autism & Developmental Medicine Institute, Geisinger, Danville, PA 17822, USA; epalen@geisinger.edu (E.P.); arheidlebaugh@geisinger.edu (A.R.H.); keholdren@geisinger.edu (K.H.); lkwalsh1@geisinger.edu (L.K.W.); mtoetjens@geisinger.edu (M.T.O.); dr.david.ledbetter@gmail.com (D.H.L.); clmartin1@geisinger.edu (C.L.M.); 2 Biomedical Ethics Unit, McGill University, Montreal, QC H3A 1X1, Canada; kasia.tolwinski@mail.mcgill.ca

**Keywords:** genomic screening, personal utility, neuropsychiatric disorders, brain disorders, copy number variant

## Abstract

Genomic variants that cause neurodevelopmental/psychiatric disorders (NPD) are relatively prevalent and highly penetrant. This study aimed to understand adults’ immediate responses to receiving NPD-related results to inform inclusion in population-based genomic screening programs. Nine recurrent, pathogenic copy number variants (CNVs) were identified from research exome data, clinically confirmed, and disclosed to adult participants of the Geisinger MyCode Community Health Initiative DiscovEHR cohort by experienced genetic counselors. A subset of in-person genetic counseling sessions (*n* = 27) were audio-recorded, transcribed, and coded using a grounded theory approach. Participant reactions were overwhelmingly positive and indicated that an NPD genetic etiology was highly valuable and personally useful. Participants frequently reported learning disabilities or other NPD that were not documented in their electronic health records and noted difficulties obtaining support for NPD needs. Most intended to share their genetic result with family members and health care providers and were interested in how their result could improve their healthcare. This study indicates that results from population-based NPD genomic screening can provide personal value for adults with NPD, were viewed positively by participants, and could improve clinical outcomes by informing symptom monitoring for NPD and co-morbidities, promoting improved health behaviors, and enhancing psychotherapeutic approaches.

## 1. Introduction

Genetic testing has been widely implemented across clinical settings and employs technologies that allow for broad assessment of genomic variation across hundreds to thousands of genes or the entire genome [1,2,3,4,5,6,7,8,9,10]. Chromosomal microarray and next-generation sequencing methods have become standard of care testing recommendations for patients with a wide variety of clinical indications, including those with neurodevelopmental/psychiatric disorders (NPD) [1,2,3,4,5]. Despite clinical guidelines and professional practice recommendations, many individuals with clear indications are not offered genetic testing, precluding care from being informed by a genetic etiology.

Recognizing the limitations of clinical guidelines in ensuring adequate genetic diagnosis, population-based genomic screening models have been developed to identify high-risk individuals using a genomics-first approach rather than waiting for symptoms, adverse medical events, or a family history to be revealed [11,12]. This proactive strategy has typically been limited to genomic disorders for which medical interventions, procedures, or medications are available to reduce morbidity and mortality, such as hereditary cancer and cardiovascular disorders. Yet, by strictly limiting the inclusion of genomic disorders to those with specific medical interventions, such programs may be denying patients access to clinically and personally meaningful genomic information. Additional critical factors that have been described for population genomic screening implementation frameworks include early disease detection or management, access to social services, implications for family members, and personal utility [13].

NPD represent an etiologically heterogenous group of clinically defined disorders, including autism spectrum disorder, intellectual disability, epilepsy, schizophrenia, bipolar disorder, and others [14,15]. These disorders are collectively common, affecting at least 14–18% of children and adults in the United States [16,17]. NPD are defined individually by clinical assessment and observation, yet they represent variable manifestations of underlying developmental brain dysfunction and share common genetic etiologies, including high-impact, pathogenic genetic variants that are amenable to identification via population genomic screening, such as recurrent, pathogenic copy number variants (CNV) and sequence-level variants [14,15]. Clinical genetic testing for patients with NPD has been embraced in pediatric settings where combined diagnostic yields for sequence variants and CNVs approach 40% in certain cohorts [1,7,18,19,20,21,22]. However, testing is rarely offered to adults with NPD, representing a significant gap in access to genetic diagnoses and a lost opportunity to proactively inform the care of family members [15].

We have previously described the estimated prevalence and penetrance of NPD-related CNVs in the DiscovEHR cohort, a subset of the Geisinger MyCode^®^ Community Health Initiative with paired exome and electronic health record (EHR) data, which exceeded or were comparable to estimates for genomic disorders traditionally included in population genomic screening programs [15]. The CNV prevalence (0.8%) and penetrance (35–70%) estimates within this unselected, health system-based population indicate that allocation of resources for inclusion of NPD in such precision health models is warranted [15]. Here, we provide the first detailed evaluation of adult individuals’ reactions to receiving results from an NPD population-based genomic screening program.

## 2. Materials and Methods

### 2.1. CNV Detection and Genetic Counseling Disclosure Process

Recurrent, segmental duplication-mediated, pathogenic CNVs were identified from exome sequencing data from the Geisinger MyCode DiscovEHR cohort, and paired EHR data were available for review. Nine CNVs (Table S1) were prioritized for disclosure based on frequency within the dataset and presence of non-NPD medical implications, as previously described [15]. All CNVs were confirmed in a CAP- and CLIA-certified laboratory prior to disclosure.

At this initial stage, CNVs were disclosed only to individuals with an NPD history, based on EHR review. Our previously described results disclosure process was based on established MyCode protocols [12,15]. Briefly, participants and their primary healthcare providers were notified by letter that a clinically relevant genetic result was identified through the MyCode research project, and participants were offered an appointment with a certified genetic counselor with significant experience with NPD-related CNVs. A detailed genetic counseling (GC) session, including explanation of the CNV, inheritance, the variable nature of clinical manifestations, and exploration of personal and family history, was available in-person or by telephone. Genetic counselors explored participants’ immediate reactions and intentions to communicate with family members and medical providers about their result. Support persons were welcome to attend. The genetic test report and GC sessions were documented in the EHR and primary healthcare providers received CNV-specific supportive medical literature.

### 2.2. Qualitative Analysis of Audio-Recorded In-Person Genetic Counseling Sessions

Participants presenting for in-person GC disclosure sessions were eligible to provide written consent or assent (IRB #2017–0273) for session audio-recording. Twenty-seven participants provided written consent. One participant (ADMI23) was able to provide assent and written consent was obtained from his mother, who is his legal guardian (ADMI24). Enrollment continued until thematic saturation was reached. This approach was utilized to collect direct participant responses to receiving NPD genetic results and evaluate responses within the context discussed in a GC disclosure setting. Recordings were transcribed verbatim. A grounded theory approach with two independent coders (KW and KT) was used to develop an initial codebook using the first 14 transcripts [15,23]. The remaining 13 transcripts were similarly coded independently and discussed to assess for thematic saturation and to refine the final codebook. No additional themes emerged upon analysis of the remaining 13 transcripts. Final codes were applied to all transcripts after consensus was reached. Participant-reported NPD diagnoses were identified by independent coders (EP and AH) and consensus was achieved. Participant-reported diagnoses were compared to participants’ EHR documentation to identify EHR documentation gaps.

## 3. Results

Of the 31 eligible participants from July 2017 to May 2019, only three declined. Twenty-seven GC session transcripts were available (Table 1). One GC session was attended by a mother and son (ADMI24 and ADMI23, respectively) who received their results together; the son was largely non-participatory due to NPD severity. Seventeen participants (60.7%) were female and the mean age was 49.9 years (range of 23–75 years).

Seven key themes emerged (Table 2) and are described below with exemplary quotes. Participants often made connections between their NPD experiences and those of family members and placed the genetic information within their family history context. Therefore, themes described below often applied to the individual and family level.

### 3.1. NPD Genetic Information Was Inherently Valuable

Participants universally described the genetic information as inherently valuable for the insight it provided into their personal and family histories and potential usefulness for family members (all 27 GC sessions). Participants wanted information about themselves simply for the sake of it: “it’s cool” (ADMI10, Male, 22q11.2 del) and “it’s piquing my curiosity” (ADMI17, Male, 16p11.2 del). One participant said, “it’s better to know than to not know” (ADMI05, Female, 1q21.1 del), which was consistent across participants. Identifying a medical explanation was important: “It feels good to know […] that there’s a name for my condition” (ADMI10, Male, 22q11.2 del). Another noted, “It’s nice to have an answer, you know? […] it gives that peace of mind” (ADMI09, Female, 16p13.11 del).

Accompanying family members shared these reactions. In one GC session, a participant’s mother was reflecting on her son’s challenges: “I wish they would have [known] all this when he was born” (ADMI06, Male, 16p11.2 del). After receiving results, many participants intended to learn more about their CNVs: “I guess it’s nice to know that I’ll probably be able to have some information that I never had my hands on before because you Google it, you look it up on the computer, and they give you all these things you can go to” (ADMI07, Female, 17q11.2 del). Information about one’s genomic make-up was considered personally useful and relevant to participants’ personal health and well-being.

Participants were typically open to discussing their result with their healthcare provider. One participant shared that he hoped it would lend some legitimacy to his anxiety complaints and lead to better care: “the last time I was in to see [his provider] for a yearly check-up she’s like, ‘Well, I don’t see the reason why you should be on anxiety medication’” (ADMI02, Male, 16p13.11 del). Plans to use their result to broach mental health discussions were echoed by others: “I think I probably will. I need to get some kind of medication changes or something that works better for me” (ADMI03, Female, 16p13.11 del). One participant wanted to educate her insurance company: “you saying to me the depression is a part of that [helps] because that’s what I can’t get through to [insurance company]. That in itself, along with something like this for me to send to them, might be very beneficial” (ADMI07, Female, 17q11.2 del).

Many participants were eager to share their results with family members, believing it could be beneficial to them. Reasons for sharing included helping those with similar NPD challenges, seeking out support for themselves, future family planning, and giving others this new context for their shared history. One participant said of her son, “I think this would help him because that way he would understand why he’s not so much book smart” and “it could help him if he has kids […] because if they’re slow in reading or slow [it could be because of] this chromosome” (ADMI05, Female, 1q21.1 del).

### 3.2. Lifelong NPD Challenges Were Discussed Openly

**Figure 1:**
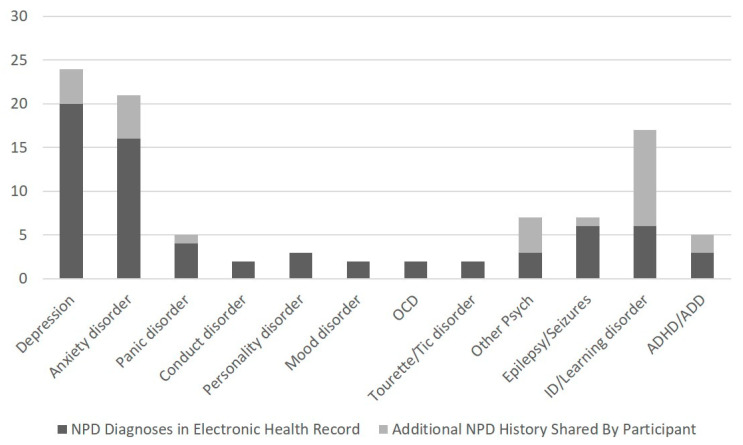
Neurodevelopmental/psychiatric diagnoses there were documented in participants’ electronic health record (EHR) versus participant reported diagnoses that were absent from the EHR. NPD—neurodevelopmental/psychiatric disorder; OCD—obsessive compulsive disorder; ID—intellectual disability; ADHD—attention deficit hyperactivity disorder; ADD—attention deficit disorder.

In all 27 GC sessions, participants openly discussed their lifelong NPD challenges, much of which were not documented in the EHR. Figure 1 illustrates the clinical NPD diagnoses documented in participants’ EHRs at the time of results disclosure and the additional NPD history shared during GC sessions. Learning disorders were the most common NPD concerns that were undocumented in EHRs; it was absent from the EHRs of 11 of the 17 individuals (64.7%) reporting this history.

Learning disabilities, interpersonal challenges, and mental health concerns were typically noted in childhood, and participants described the impact of NPD as adults, particularly in the workplace.

“I was a slow learner, I know that, even in high school I can remember that. I didn’t comprehend quickly. Things just didn’t sink in and they still don’t. I just have a hard time sitting still for a very long time and trying to comprehend what it is I’m reading.”

(ADMI07, Female, 17q11.2 del)

“There’s other times where I would just snap just for no reason, just literally just go off the deep, just something would just hit me the wrong way and I would just go off for no reason. Then after the fact it would bother me and really upset me to where I would actually shut down myself and just stay away from people. […] I lost several jobs because of [depression and alcoholism].”

(ADMI02, Male, 16p13.11 del)

“At work I get mad because one of my coworkers, something’s wrong or whatever, I’m trying to cope with it because I don’t want to go off on someone the wrong way and ruin it, get fired, or ruin a relationship or whatever.”

(ADMI06, Male, 16p11.2 del)

These challenges sometimes resulted in regret and feelings of inadequacy, and a lack of support was a common experience.

“When I was in school they just went, ‘You have [ADHD],’ and sent me away. It took a long time to get medicine because doctors back then didn’t really want to prescribe.”

(ADMI18, Male, 15q13.3 del)

“Oh, when I hear about other women that have been in the same [social situation] that have gone out and started their own companies and done things like that, I feel really inadequate, and what I want to do more than anything is make my kids proud, and I’m not doing that, and I want to show them that it is possible to rise from the ashes. I don’t see a way of doing that, because I can’t go to school because I’m not cut out for it.”

(ADMI01, Female, 16p13.11 del)

### 3.3. NPD Genetic Information Fit with Participants’ Lived Experiences

The CNV diagnosis provided a new, medical explanation for participants’ self-disclosed learning, physical, social, or mental health challenges, and they frequently reported that the result “made sense” (23 GC sessions). Participants identified with CNV natural history descriptions, often saying, “I have that!” (ADMI20, Male, 16p11.2 del). The natural histories fit with what they knew about themselves: “Look at this [information on CNV]! Right here. One, two, three. Too late in learning to sit, move, walk. Too late in starting to speak in language. Children may need support with learning […] That was me. That was me!” (ADMI24, Female, 16p13.11 del). These were often positive “eureka” moments when the participant made connections between their life experiences and the genetic etiology as a new framework for self-understanding.

One participant shared “[this result] just confirmed what I’ve known all years of my life. […] I knew back when I was growing up there was something that just wasn’t right. I knew I had to be a little bit different somehow” (ADMI14, Female, 16p11.2 del). Another participant said, “Ah, so that explains it,” and later spoke of the relief she felt: “knowing that it’s not just in my head, I’m just an anxious person” (ADMI12, Female, 1q21.1 del). For this participant, recognizing a medical or biological explanation provided a sense of relief and alleviated guilt. Another participant reflected on his tendency for emotional outbursts: “It was just something I dealt with, my way of dealing with things. I just accepted that my whole life, but now seeing this, like I said, a lot of things that went on kind of makes sense” (ADMI02, Male, 16p13.11 del). While some participants reported that their results led to new self-understanding, we did not observe a negative impact to participants’ sense of self. For example, when asked if the result made him feel differently, this participant said, “Not really. It’s still the same problem, just explained why I could have it” (ADMI18, Male, 15q13.3 del).

Furthermore, NPD genetic results provided insight into physical symptoms, like extreme obesity associated with 16p11.2 deletion and clinical features of neurofibromatosis caused by 17q11.2 deletion. One participant expressed understanding that weight loss “is going to be harder for me” and shared that “it’s starting to explain some things to me […] the seizures, my weight. […] I am just happy that it finally begins to answer why I do some of the things I do” (ADMI17, Male, 16p11.2 del).

### 3.4. Previously Held Causal Attributions for Personal and Family NPD Histories Were Common

Most participants stated they had previously attributed their NPD diagnoses or challenges to an explanation or rationale, especially social situations or experiences (26 GC sessions). This view was the lens through which they had understood these challenges. Participants often attributed childhood challenges to moving or parents divorcing. Job-related stress was mentioned as a major contributor to mental health concerns, as were divorce, deaths in the family, and traumatic life events in adulthood.

Some participants described their NPD histories in familial terms: “the depression and anxiety is what most of the people in the family have […]” (ADMI18, Male, 15q13.3 del). Past illnesses or medical procedures, such as stroke, injuries from surgery, lack of oxygen at birth, unrelated genetic conditions, and viruses were also described as NPD attributions.

### 3.5. Negative Emotions Were Less Prominent than Positive Emotions and Were Associated with NPD-Related Lived Experiences Rather than the Genetic Result

Participants expressed both positive and negative feelings about receiving their NPD genetic result, though positive feelings predominated (26 GC sessions), and the majority described their experience as positive. Negative feelings were expressed in 13 of the 27 GC sessions (48.1%) but, of these 13, positive emotions were also expressed in 12 GC sessions (92.3%). Some individuals described initial anxiety upon receiving their notification letter because they thought the information might be cancer-related. However, they stated that this anxiety was alleviated after talking with a genetic counselor: “I’m just glad it’s nothing serious” (ADMI11, Male, 16p11.2 del).

Negative emotions, including sadness, anger, and frustration, emerged when participants discussed their NPD-related struggles and were not in direct response to the genetic result. One participant preferred not to discuss his psychiatric hospitalization because it was painful to revisit. Other participants expressed worry about the well-being of family members, particularly children. Worry often evolved into hope during the sessions, with participants envisioning a different future for younger relatives, one with more support and less judgment.

Only one participant expressed only negative emotions triggered by his childhood: “now that I’m talking about it, a little bit upset, I feel like I want to just break down and cry” (ADMI02, Male, 16p13.11 del). The genetic counselor followed up with him multiple times to offer support and assess his state. He was consistently stable, reported improvement, and was taking active steps toward establishing care with a therapist. Despite his feelings, this participant thought the information could be valuable for his teenage son who had significant behavioral problems: “talking about this I see certain aspects in my son’s life [in] different ways. He acts and lashes out and I’m hoping this doesn’t affect him. If it does, I want to know ways to help him deal with it because I don’t want him having to go through what I went through” (ADMI02, Male, 16p13.11 del).

### 3.6. NPD Genetic Information Was Received with a Resilient Attitude

Despite NPD affecting many aspects of participants’ lives, a resilient attitude was a common reaction to receiving genetic results (17 GC sessions). Participants felt that they had the ability to cope or overcome challenges, often with help from their families.

“I mean we’re comfortable with who we are and we’re old enough to not be overly worried. We’re all self-sufficient. We can do life. We were smart outside of school, so I quit and I enrolled in cosmetology school. Like I said, I did really well.”

(ADMI01, Female, 16p13.11 del)

“You just have to know your own barriers to jump over that hurdle to see if you can broaden it so you can learn how you learn.”

(ADMI06, Male, 16p11.2 del)

This resilience was also expressed by caregivers of individuals with NPD. Said one mother of her son: “I got him. I just kept going and going.” (ADMI24, Female, 16p13.11). Another participant reflected, “in retrospect now, I’m glad I wasn’t too forceful [with schooling], because if there is this issue, that would’ve just made them miserable, so I’m glad I was not super strict on that, because she’s got friends” (ADMI01, Female, 16p13.11 del). These participants appeared realistic about their children’s abilities, encouraging them in areas where they might find success.

### 3.7. Interest in the Implications of NPD Genetic Information on Clinical Management

Participants’ questions were frequently related to NPD management options (17 GC sessions). Many participants explicitly asked if the CNV could be corrected: “I want to know how we can fix it” (ADMI05, Female, 1q21.1 del), and “is there a cure for it?” (ADMI20, Male, 16p11.2 del). They were eager to use the information to improve their NPD care and asked if medications could target their specific CNV: “There’s no special type of medication that will help the depression now that we know?” (ADMI18, Male, 15q13.3 del).

Participants also asked about future illnesses and impacts: “My last question is, what does this mean for me in the future, […] how is it going to affect me for the rest of my life?” (ADMI01, Female, 16p13.11 del). Several participants had experienced CNV-related medical concerns, such as seizures, obesity, and tumors. Most were relieved that they would not likely face new CNV-related problems, which did not diminish the information’s value. Participants wanted their healthcare providers to know about CNV-associated health risks, such as renal cysts and maturity-onset diabetes of the young caused by 17q12 deletions [24], and for their test result and medical implications to be documented in the EHR.

## 4. Discussion

Population-based genomic screening aims to identify individuals with clinically relevant genomic variants without the bias of relying on clinically defined diagnoses and before symptoms develop or progress in severity [11,12,15,25,26,27]. Such programs could be particularly valuable for adults with NPD since clinical genetic testing utilization in adult NPD settings has lagged behind other medical specialties, resulting in an access gap for genetics-informed clinical care. This gap may be due in part to a perception that psychiatric illnesses are only associated with common, polygenic risk factors, rather than high-impact, rare pathogenic genomic variants that are causative for brain disorders manifesting with marked variability [28]. Concern has also been raised that learning of NPD genomic information could harm patients through increased anxiety, a negative impact on self-image, or decreased agency over one’s mental health needs [29].

This study sought to explore adult individuals’ immediate reactions to receiving NPD results through population-based genomic screening, with a focus on nine pathogenic CNVs that are relatively prevalent, penetrant throughout the lifespan, and not often readily diagnosed by clinical features [15]. This is an important differentiation from NPD genetic disorders that are easily recognized, such as Down syndrome, and neurodegenerative genetic disorders where testing is available to asymptomatic at-risk individuals. Our findings indicate that participants’ immediate reactions were generally positive, with little cause for concern for immediate negative reactions to the genetic information itself. Even in the context of difficult memories, participants overwhelmingly stated that they were glad to have their genetic result, that it was valuable information which “medicalized” their NPD lived experiences, and that they would prefer to know than to not know. This is consistent with data that indicate public interest in receiving genomic results for neurological disorders, regardless of severity or treatment options [30].

Genomic information is generally considered to have personal utility if “it can reasonably be used for decisions, actions or self-understanding which are personal in nature” [31]. Evidence of personal utility is strongly present in this dataset as NPD genetic results were considered inherently valuable because of their personal nature and ability to shed light on lived experiences. NPD genetic results were assimilated into participants’ personal narratives, helping them understand past difficulties or events and sometimes prompting a re-evaluation of previously held beliefs or decisions about seeking support.

Population-based NPD genomic screening may provide participants with an opportunity to re-evaluate the symptom-based care they are receiving or of which they are in need. Our experience suggests that participants were appreciative of the opportunity to explore their NPD history in the context of these causal genomic variants, and we hypothesize that NPD genetic etiologies could promote heightened awareness of mental health and be incorporated into psychological therapies. Furthermore, participants frequently expressed reassurance that they were not at fault for their NPD and that their “sense of self” either did not change or improved. These sentiments were motivators for discussing their genetic result with family members, as opposed to barriers. These results indicate that knowledge of causal NPD variants could reduce the impacts of stigma associated with NPD symptoms and clinical diagnoses by providing a medical explanation and unifying NPD experiences.

Population-based NPD genomic screening is a promising model to provide individuals access to clinically and personally useful NPD etiologies without relying on an inadequate symptom-based referral system. Certified genetic counselors are critical to these programs to promote participants’ adaptation to and understanding of results and to support positive health behaviors, while exploring family dynamics and fostering communication and mutual support within the family. Armed with this information, medically indicated referrals can be made (e.g., monitor for hypocalcemia for adults with 22q11.2 del) [32], attention can be paid to mental health and well-being concerns, and familial cascade testing can be offered.

### Study Limitations

This study does not provide longitudinal data regarding participants’ perceptions and does not address participants’ actual communication and health behavior choices. Follow-up studies which assess for negative longer-term consequences and actual health behaviors in response to results are currently in progress and could identify opportunities to address participant and healthcare provider support needs proactively. These data only include participants who had clinical documentation of NPD in their EHR and who had one of nine pathogenic CNVs. Thus, individuals who do not have clinical NPD diagnoses may respond differently or report less personal utility. We have begun returning NPD results to individuals regardless of the NPD documentation in their EHR and preliminary findings indicate similar responses. Our results are also biased towards individuals who responded to invitations to meet with a genetic counselor and preferred an in-person appointment. Therefore, generalization of our results to all individuals who may receive an NPD genetic etiology is limited and studies that include broader participants and longitudinal approaches are warranted.

## 5. Conclusions

This is the first study to report a detailed description of adults’ responses to receiving NPD genetic diagnoses through population-based NPD genomic screening. Responses were overall positive, and the genomic information was considered highly valuable. Our findings strongly indicate that a genetic diagnosis could improve an individual’s perception of their NPD history and promote communication with supportive family members and healthcare providers. Proactive identification of NPD genetic etiologies could provide additional clinical value by promoting improved health behaviors and adherence to treatment recommendations or could be incorporated into individual or family-based support services and/or therapies.

## Figures and Tables

**Table 1:** Adult participant demographics and NPD diagnoses from the EHR.

Study ID	Deletion	Sex	Age in Years at Disclosure	NPD Diagnoses in EHR
ADMI01	16p13.11	F	49	Anxiety, Depressive disorder
ADMI02	16p13.11	M	49	Generalized anxiety disorder, Panic disorder without agoraphobia, Adjustment disorder with anxiety, Depressive disorder
ADMI03	16p13.11	F	63	Adjustment disorder with depression, Depressive disorder
ADMI04	16p11.2	M	70	Adjustment disorder, Intellectual disability
ADMI05	1q21.1	F	51	Anxiety, Adjustment disorder with depressed mood, Depressive disorder
ADMI06	16p11.2	M	23	Unspecified disturbance of conduct, ADHD, Other specific developmental learning difficulties, Unspecified delay in development
ADMI07	17q11.2	F	54	Major depressive affective disorder-recurrent, Unspecified episodic mood disorder, Anxiety, Adjustment disorder with depressed mood, Depressive disorder, ADD
ADMI08	16p11.2	F	56	Adjustment disorder with depressed mood, Depressive disorder
ADMI09	16p13.11	F	42	Major depressive affective disorder-recurrent episode-severe-specified as with psychotic behavior, Borderline personality disorder, Depressive disorder
ADMI10	22q11.2	M	34	Unspecified intellectual disability, Mild cognitive impairment, Epilepsy
ADMI11	16p11.2	M	62	Anxiety, Depressive disorder
ADMI12	1q21.1	F	55	Generalized anxiety disorder
ADMI13	16p11.2	F	29	Depressive disorder
ADMI14	16p11.2	F	52	Generalized anxiety disorder, Depressive disorder
ADMI15	1q21.1	F	61	Depression
ADMI16	17q12	F	32	Mood disorder, Adjustment disorder, Panic disorder
ADMI17	16p11.2	M	44	Mild intellectual disability, Epilepsy, Panic disorder
ADMI18	15q13.3	M	37	Anxiety, Major depressive affective disorder, Epilepsy
ADMI19	16p11.2	M	49	Convulsive epilepsy
ADMI20	16p11.2	M	37	Mild cognitive impairment, Generalized anxiety disorder, Obsessive compulsive disorder, Tourette’s, Major depressive disorder, Disturbance of conduct
ADMI21	16p11.2	M	75	Anxiety, Major depression, Acute reaction to stress
ADMI22	16p11.2	F	55	Depression, Anxiety
ADMI23	16p13.11	M	24	Unspecified intellectual disabilities, Delayed milestones, Epilepsy, Speech/language disorder, ADHD, Anxiety, Obsessive-compulsive disorder, Tic disorder
ADMI24	16p13.11	F	56	Generalized anxiety disorder, Depression
ADMI25	16p13.11	F	27	Anxiety, Epilepsy, Adjustment disorder, Chronic depressive personality disorder, Major depressive disorder
ADMI26	16p13.11	F	33	Adjustment disorder, Depression, Dysthymic disorder, Generalized anxiety disorder, Bipolar disorder
ADMI27	16p13.11	F	53	Major depressive disorder, Borderline personality disorder, Generalized anxiety disorder
ADMI28	16p13.11	F	69	Depression, Panic disorder

NPD—neurodevelopmental/psychiatric disorder; EHR—electronic health record; F—female; M—male; ADHD—attention deficit hyperactivity disorder; ADD—attention deficit disorder.

**Table 2:** Seven main themes representing participant responses to receiving NPD-related CNVs from 27 genetic counseling sessions.

Theme Description
NPD genomic information was inherently valuable (27 sessions)
Lifelong NPD challenges were discussed openly (27 sessions)
NPD genomic information fit with participants’ lived experiences (23 sessions)
Previously held causal attributions for personal and family NPD histories were common (26 sessions)
Negative emotions were less prominent than positive emotions and were associated with NPD-related lived experience rather than the genetic result (26 sessions)
NPD genomic information was received with a resilient attitude (17 sessions)
Interest in the implications of NPD genomic information on clinical management (17 sessions)

NPD—neurodevelopmental/psychiatric disorder; CNV—copy number variant.

## Data Availability

De-identified data presented in this study are available on request from the corresponding author.

## Supplementary Materials

The following are available online at https://www.mdpi.com/article/10.3390/jpm11050365/s1, Table S1: Inclusion Criteria and Genomic Coordinates Used to Indicate Copy Number Variant Confirmation Testing.

## References

[1] Miller D.T., Adam M.P., Aradhya S., Biesecker L.G., Brothman A.R., Carter N.P., Church D.M., Crolla J.A., Eichler E.E., Epstein C.J. (2010). Consensus Statement: Chromosomal Microarray Is a First-Tier Clinical Diagnostic Test for Individuals with Developmental Disabilities or Congenital Anomalies. Am. J. Hum. Genet..

[2] ACMG Board of Directors (2012). Points to consider in the clinical application of genomic sequencing. Genet. Med..

[3] Yang Y., Muzny D.M., Reid J.G., Bainbridge M.N., Willis A., Ward P.A., Braxton A., Beuten J., Xia F., Niu Z. (2013). Clinical Whole-Exome Sequencing for the Diagnosis of Mendelian Disorders. N. Engl. J. Med..

[4] Lee H., Deignan J.L., Dorrani N., Strom S.P., Kantarci S., Quintero-Rivera F., Das K., Toy T., Harry B., Yourshaw M. (2014). Clinical Exome Sequencing for Genetic Identification of Rare Mendelian Disorders. JAMA.

[5] Farwell K.D., Shahmirzadi L., El-Khechen D., Powis Z., Chao E.C., Davis B.T., Baxter R.M., Zeng W., Mroske C., Parra M.C. (2015). Enhanced utility of family-centered diagnostic exome sequencing with inheritance model-based analysis: Results from 500 unselected families with undiagnosed genetic conditions. Genet. Med..

[6] Wooderchak-Donahue W., VanSant-Webb C., Tvrdik T., Plant P., Lewis T., Stocks J., Raney J.A., Meyers L., Berg A., Rope A.F. (2015). Clinical utility of a next generation sequencing panel assay for Marfan and Marfan-like syndromes featuring aortopathy. Am. J. Med. Genet..

[7] Retterer K., Juusola J., Cho M.T., Vitazka P., Millan F., Gibellini F., Vertino-Bell A., Smaoui N., Neidich J., Monaghan K.G. (2016). Clinical application of whole-exome sequencing across clinical indications. Genet. Med..

[8] Hooker G.W., Clemens K.R., Quillin J., Postula K.J.V., Summerour P., Nagy R., Buchanan A.H. (2017). Cancer Genetic Counseling and Testing in an Era of Rapid Change. J. Genet. Couns..

[9] Posey J.E., Harel T., Liu P., Rosenfeld J.A., James R.A., Akdemir Z.H.C., Walkiewicz M., Bi W., Xiao R., Ding Y. (2017). Resolution of Disease Phenotypes Resulting from Multilocus Genomic Variation. N. Engl. J. Med..

[10] Hershberger R.E., Givertz M.M., Ho C.Y., Judge D.P., Kantor P.F., McBride K.L., Morales A., Taylor M.R., Vatta M., Ware S.M. (2018). Genetic Evaluation of Cardiomyopathy—A Heart Failure Society of America Practice Guideline. J. Card. Fail..

[11] Gray S.W., Martins Y., Feuerman L.Z., Bernhardt B.A., Biesecker B.B., Christensen K.D., Joffe S., Rini C., Veenstra D., Members of the CSER Consortium Outcomes and Measures Working Group (2014). Social and behavioral research in genomic sequencing: Approaches from the Clinical Sequencing Exploratory Research Consortium Outcomes and Measures Working Group. Genet. Med..

[12] Williams M.S., Buchanan A.H., Davis F.D., Faucett W.A., Hallquist M.L.G., Leader J.B., Martin C.L., McCormick C.Z., Meyer M.N., Murray M.F. (2018). Patient-Centered Precision Health in A Learning Health Care System: Geisinger’s Genomic Medicine Experience. Health Aff..

[13] Chanfreau-Coffinier C., Peredo J., Russell M.M., Yano E.M., Hamilton A.B., Lerner B., Provenzale D., Knight S.J., Voils C.I., Scheuner M.T. (2018). A logic model for precision medicine implementation informed by stakeholder views and implementation science. Genet. Med..

[14] Moreno-De-Luca A., Myers S.M., Challman T.D., Moreno-De-Luca D., Evans D.W., Ledbetter D.H. (2013). Developmental brain dysfunction: Revival and expansion of old concepts based on new genetic evidence. Lancet Neurol..

[15] Martin C.L., Wain K.E., Oetjens M.T., Tolwinski K., Palen E., Hare-Harris A., Habegger L., Maxwell E.K., Reid J.G., Walsh L.K. (2020). Identification of Neuropsychiatric Copy Number Variants in a Health Care System Population. JAMA Psychiatry.

[16] Zablotsky B., Black L.I., Maenner M.J., Schieve L.A., Danielson M.L., Bitsko R.H., Blumberg S.J., Kogan M.D., Boyle C.A. (2019). Prevalence and Trends of Developmental Disabilities among Children in the United States: 2009–2017. Pediatrics.

[17] Any Mental Illness (AMI) Among Adults. (n.d.). http://www.nimh.nih.gov/health/statistics/prevalence/any-mental-illness-ami-among-adults.shtml.

[18] Srivastava S., Love-Nichols J.A., Dies K.A., Ledbetter D.H., Martin C.L., Chung W.K., Firth H.V., Frazier T., Hansen R.L., the NDD Exome Scoping Review Work Group (2019). Meta-analysis and multidisciplinary consensus statement: Exome sequencing is a first-tier clinical diagnostic test for individuals with neurodevelopmental disorders. Genet. Med..

[19] Hochstenbach R., Buizer-Voskamp J., Vorstman J., Ophoff R. (2011). Genome Arrays for the Detection of Copy Number Variations in Idiopathic Mental Retardation, Idiopathic Generalized Epilepsy and Neuropsychiatric Disorders: Lessons for Diagnostic Workflow and Research. Cytogenet. Genome Res..

[20] Clark M.M., Stark Z., Farnaes L., Tan T.Y., White S.M., Dimmock D., Kingsmore S.F. (2018). Meta-analysis of the diagnostic and clinical utility of genome and exome sequencing and chromosomal microarray in children with suspected genetic diseases. NPJ Genom. Med..

[21] Vissers L.E.L.M., Gilissen C., Veltman J.A. (2016). Genetic studies in intellectual disability and related disorders. Nat. Rev. Genet..

[22] Trujillano D., Bertoli-Avella A.M., Kandaswamy K.K., Weiss M.E., Köster J., Marais A., Paknia O., Schröder R., Garcia-Aznar J.M., Werber M. (2017). Clinical exome sequencing: Results from 2819 samples reflecting 1000 families. Eur. J. Hum. Genet..

[23] Timmermans S., Tavory I. (2012). Theory Construction in Qualitative Research: From Grounded Theory to Abductive Analysis. Sociol. Theory.

[24] Mitchel M.W., Moreno-De-Luca D., Myers S.M., Levy R.V., Turner S., Ledbetter D.H., Martin C.L., Adam M.P., Ardinger H.H., Pagon R.A., Wallace S.E. (1993–2021). 17q12 Recurrent Deletion Syndrome. GeneReviews [Internet].

[25] Gabai-Kapara E., Lahad A., Kaufman B., Friedman E., Segev S., Renbaum P., Beeri R., Gal M., Grinshpun-Cohen J., Djemal K. (2014). Population-based screening for breast and ovarian cancer risk due to *BRCA1* and *BRCA2*. Proc. Natl. Acad. Sci. USA.

[26] Vassy J.L., Christensen K.D., Schonman E.F., Blout C.L., Robinson J.O., Krier J.B., Diamond P.M., Lebo M., Machini K., Azzariti D.R. (2017). The impact of whole-genome sequencing on the primary care and outcomes of healthy adult patients: A pilot randomized trial. Ann. Intern. Med..

[27] Buchanan A.H., Manickam K., Meyer M.N., Wagner J.K., Hallquist M.L.G., Williams J.L., Rahm A.K., Williams M.S., Chen Z.M., Shah C.K. (2018). Early cancer diagnoses through BRCA1/2 screening of unselected adult biobank participants. Genet. Med..

[28] Dubovsky S.L. (2016). The Limitations of Genetic Testing in Psychiatry. Psychother. Psychosom..

[29] Lebowitz M.S. (2014). Biological conceptualizations of mental disorders among affected individuals: A review of correlates and consequences. Clin. Psychol. Sci. Pract..

[30] Graves K.D., Sinicrope P.S., McCormick J.B., Zhou Y., Vadaparampil S.T., Lindor N.M. (2015). Public Perceptions of Disease Severity but Not Actionability Correlate with Interest in Receiving Genomic Results: Nonalignment with Current Trends in Practice. Public Health Genom..

[31] Bunnik E.M., Janssens A.C.J.W., Schermer M.H.N. (2014). Personal utility in genomic testing: Is there such a thing?. J. Med. Ethics.

[32] Fung W.L.A., Butcher N.J., Costain G., Andrade D.M., Boot E., Chow E.W., Chung B., Cytrynbaum C., Faghfoury H., Fishman L. (2015). Practical guidelines for managing adults with 22q11.2 deletion syndrome. Genet. Med..

