# Supplementary material for: Population Genomic Screening for Genetic Etiologies of Neurodevelopmental/Psychiatric Disorders Demonstrates Personal Utility and Positive Participant Responses
Source: J Pers Med. 2021 May 1;11(5):365. doi: 10.3390/jpm11050365 (PMC8147408; doi:10.3390/jpm11050365)
Supplement: Supplementary file 1 [file jpm-11-00365-s001.zip › jpm-1182757-supplementary.pdf]

Table S1: Inclusion Criteria and Genomic Coordinates Used to Indicate Copy Number Variant Confirmation Testing

**Copy Number Variant**	**Criteria**	**Genomic Coordinates (GRCh38/hg38; Mb)**
1q21.1 (*GJA5*) del*	Size >50% of critical region	chr1:147.11-147.92
1q21.1 (*GJA5* + TAR) del*	Size >50% of critical region	chr1:145.63-147.92
7q11.23 (*ELN*) del	Size >50% of critical region	chr7:73.33-74.73
15q13.3 (*CHRNA7*) del	Size >50% of critical region	chr15:30.84-32.15
15q24 (*SIN3A*) del	At least 1 Mb between the A-E intervals	chr15:72.67-75.68
16p13.11 (*MYH11*) del	Size >50% of critical region	chr16:15.42-16.20
16p11.2 (*TBX6*) del	Size >50% of critical region	chr16:29.64-30.19
17q11.2 (*NF1*) del	Size >50% of critical region, affecting *NF1*	chr17:30.78-31.94
17q12 (*HNF1B*) del	Size >50% of critical region	chr17:36.46-37.85
22q11.2 del (proximal, A-B)#	Size >50% of critical region	chr22:18.92-20.29
22q11.2 del (proximal, A-D)#	Size >50% of critical region	chr22:18.92-21.11

* - included as 1q21.1 deletions; # - included as 22q11.2 deletions
